# Prevalence and associated factors of chronic kidney disease among diabetes mellitus patients in Ethiopia: A systematic review and meta-analysis

**DOI:** 10.1371/journal.pone.0315529

**Published:** 2025-03-05

**Authors:** Habtamu Wagnew Abuhay, Melaku Kindie Yenit, Mihret Melese, Gebrie Getu Alemu, Fantu Mamo Aragaw

**Affiliations:** 1 Department of Epidemiology and Biostatistics, Institute of Public Health, College of Medicine and Health Sciences, University of Gondar, Gondar, Ethiopia; 2 School of Health and Medical Sciences, Centre for Health Research, University of Southern Queensland, Toowoomba, Queensland, Australia; 3 Department of Human Physiology, School of Medicine, College of Medicine and Health Science, University of Gondar, Gondar, Ethiopia; Johns Hopkins University School of Medicine, UNITED STATES OF AMERICA

## Abstract

**Introduction:**

Chronic kidney disease (CKD) is a major public health concern worldwide, especially among people with diabetes mellitus (DM), which is the main cause of morbidity and mortality. In Ethiopia, the burden of CKD on DM patients is compounded by a variety of socioeconomic and healthcare-related issues. Despite the increased risk of developing CKD in DM patients, comprehensive studies on the prevalence and associated factors of CKD in this population are rare. To address this gap, this study aimed to estimate the pooled prevalence and associated factors of CKD among DM patients in Ethiopia.

**Methods:**

This systematic review and meta-analysis was carried out through an investigation of published studies in Ethiopia. A systematic literature search was performed using electronic databases such as PubMed, EMBASE, Scopus, and Google Scholar. A random-effects model was used to estimate the pooled prevalence of CKD and the odds ratio (OR) with a 95% confidence interval. The I² statistic was used to measure heterogeneity among the included studies, with a p-value < 0.05 indicating statistical significance. Publication bias was evaluated via a funnel plot and Egger’s test, and subgroup, sensitivity, and meta-regression analyses were also performed.

**Results:**

Out of the 19 included studies, the estimated pooled prevalence of CKD among DM patients in Ethiopia was 18% (95% CI 14.0, 22.0). In addition, age ≥ 60 years (OR = 3.07, 95% CI: 2.44, 3.87), rural residence (OR = 1.40, 95% CI: 1.01, 1.95), duration of DM > 5 years (OR = 2.47, 95% CI: 1.62, 3.77), proteinuria (OR = 3.30, 95% CI: 2.23, 4.88), HDL-C level < 40 mg/dL (OR = 3.08, 95% CI: 2.28, 4.16), and family history of CKD (OR = 2.58, 95% CI: 1.62, 4.09) were factors significantly associated with the prevalence of CKD among DM patients.

**Conclusion:**

The prevalence of CKD in Ethiopia was high, affecting nearly two in five individuals with diabetes. In addition, factors such as older age, rural residence, longer DM duration, positive proteinuria, lower HDL-C levels, and a family history of CKD were significantly associated with CKD prevalence. Therefore, targeted public health interventions, such as screening, education, and awareness programs, are highly recommended to mitigate this problem.

**Systematic review registrations:**

PROSPERO (2024: CRD42024576958).

## Introduction

Chronic kidney disease (CKD) is a major global public health concern, affecting about 10% of the global population, or more than 800 million people [1]. The risk is markedly higher in individuals with diabetes mellitus (DM) [2]. If left untreated, CKD results in renal failure and end-stage renal disease (ESRD) [3].

The global prevalence of CKD varies widely across different regions. Among individuals with DM, the overall global prevalence of CKD is 27% [4], while in Middle Eastern countries, it rises to 29% [5]. In Africa, there has been a notable increase in non-communicable diseases (NCDs), particularly diabetes [6]. As a result, the prevalence of CKD in this region ranges from 11% to 83.7% [7]. The burden of CKD was increasing due to the rising prevalence of diabetes mellitus [4]. CKD leads to significant morbidity and mortality and results in substantial economic burdens on healthcare systems worldwide [8]. In Ethiopia, CKD represents a significant public health issue, the prevalence of which ranges from 10% [9] to 35% [10], with increasing prevalence attributed to the growing burden of non-communicable diseases, particularly diabetes mellitus [11].

Several primary studies showed that differences between urban and rural locations, low income, lower educational status, and differences in healthcare access and socioeconomic status all contribute to the prevalence of CKD among DM patients in Ethiopia [9,12–16]. Furthermore, cultural beliefs and customs impact the health-seeking behaviors and awareness of DM patients [17] leading to delayed diagnosis and treatment of CKD. Understanding the pooled effect of these factors is crucial for developing targeted public health interventions to improve CKD prevention and management strategies. Therefore, this systematic review and meta-analysis aimed to provide a comprehensive overview of the estimated pooled prevalence of chronic kidney disease among diabetes mellitus patients, and investigates the associations between various sociodemographic, socioeconomic, and clinical factors and the prevalence of CKD among DM patients in Ethiopia. By synthesizing the available evidence, this study seeks to fill knowledge gaps, inform healthcare policymakers, and guide interventions to address the increasing burden of CKD and improve outcomes for affected individuals.

### Review questions

What is the estimated pooled prevalence of chronic kidney disease among diabetes mellitus patients in Ethiopia? What factors are associated with the prevalence of chronic kidney disease among diabetes mellitus patients in Ethiopia?

## Methods

### Protocol and registration

This systematic review and meta-analysis was registered in the international database of the Prospective Register of Systematic Reviews (PROSPERO) with registration number **CRD**42024576958. The reporting of this systematic review and meta-analysis followed the Preferred Reporting Items for Systematic Reviews and Meta-Analyses (PRISMA 2020) guidelines [18].

### Eligibility criteria

All observational studies, including cross-sectional, cohort, and case-control designs reporting the prevalence or incidence of chronic kidney disease among diabetic mellitus patients among adults aged > 18 years and who had DM, were included. All studies conducted in Ethiopia and published in English were considered in this study. Review studies, case reports, letters to the editor, articles without a full text, studies that did not provide information on the prevalence of CKD, those that involved patients with diabetes other than type 1 or type 2, and qualitative research were excluded from the review.

### Study design and search strategy

A systematic review and meta-analysis was conducted using published articles on the prevalence or incidence and associated factors of CKD among DM patients in Ethiopia.

A comprehensive search of electronic databases, including PubMed, EMBASE, Scopus, and Google Scholar, was conducted to identify relevant studies. Furthermore, the reference lists of relevant articles were examined in search of additional studies.

The search was conducted via a combination of keywords with the Boolean operators “OR” and “AND” and Medical Subject Headings (MeSH) terms related to CKD and DM. For example, to search PubMed, the following combination was used: ((Diabetes OR “diabetes mellitus” OR “Diabetes Mellitus”[Mesh] OR “type 1 diabetes” OR “type 1 diabetes mellitus” OR T1DM OR “type 2 diabetes” OR “type 2 diabetes mellitus” OR T2DM) AND (“Chronic kidney disease” OR “diabetic nephropathy” OR “Diabetic Nephropathies”[Mesh] OR “chronic renal failure” OR “renal impairment” OR proteinuria OR “end-stage kidney disease” OR “end-stage renal disease” OR “renal insufficiency”) AND (Ethiopia OR Tigray OR Afar OR Amhara OR “Benishangul-Gumuz” OR Gambela OR Harari OR Oromia OR Sidama OR Somali OR “Southern Nations Nationalities and Peoples” OR “Addis Ababa” OR “Dire Dawa”)).

The search included all published peer-reviewed articles published from the inception of the database to August 15, 2024. See (S1 Table in S2 File).

### Variable definition and measurement

This SRMA has two outcome variables. The first outcome is the prevalence of CKD, defined as an estimated glomerular filtration rate (eGFR) < 60 ml/min/1.73 m², as estimated by the Cockcroft-Gault equation. The Cockcroft-Gault equation was used to estimate the eGFR on the basis of serum creatinine levels, age, weight, and sex [19,20]. This definition was used to ensure uniformity across the studies included in this systematic review and meta-analysis.

The secondary outcome of this study was to determine the factors associated with chronic kidney disease among DM patients. To determine these associations, we utilized odds ratios (ORs) to quantify the relationship between CKD and a range of predictor variables, including age, sex, residence, family history of CKD, income level, geographical region, type of DM, duration of DM, presence of proteinuria, high-density lipoprotein cholesterol (HDL-C), and low-density lipoprotein cholesterol (LDL-C).

### Study selection and quality assessment

Relevant papers identified from the aforementioned databases and websites were imported into EndNote X9; after that, duplicate studies were removed. After the titles and abstracts were reviewed, the remaining articles were screened by two authors (MM, GGA). The full texts were independently reviewed by two authors (HWA and MKY). Any disagreements between the reviewers were resolved through further discussion involving an additional third reviewer (FMA). The authors (HWA, MKY, and FMA) subsequently evaluated the eligibility of all the retrieved studies via the Joanna Briggs Institute (JBI) quality appraisal checklist [21]. Finally, studies that scored 50% on or above the quality assessment checklist criteria were considered to be of high quality and were included in this study (S2 and S3 Tables in S2 File).

### Data extraction

Using a Microsoft Excel spreadsheet and the JBI data extraction form [22], two authors (HWA and MM) independently extracted the following information: author name, year of publication, study area, study design, sampling strategy, outcome ascertained, total participants, number of CKD cases, prevalence, and HDL-c level. Discrepancies were resolved through discussion with a third author (FMA) (S1 File).

### Statistical analysis

The statistical analysis was carried out using STATA version 17 statistical software. The relationships between factors and the prevalence of CKD were reported using the ORs. We checked for publication bias using a funnel plot and, more objectively, through Egger’s regression test [23]. There was publication bias as a result, the trim-and-fill technique was used. The heterogeneity of the studies was quantified using the I-squared statistic, and studies were classified as having low (25%), moderate (50%), or high (75%) degrees of heterogeneity [24]. A weighted random-effects model was fitted for the pooled prevalence of CKD among DM patients [25]. Subgroup analysis was performed based on the study design, type of DM, and sample size of the primary study. Finally, sensitivity analysis and meta-regression were carried out to ascertain the impact of a single study on the total estimates and to pinpoint the source of heterogeneity.

### Ethical approval and consent

Ethical approval and consent to participate were not applicable for this study, as it was a systematic review and meta-analysis based on primary studies published on chronic kidney disease in Ethiopia.

## Results

### Search results and included studies

**Fig 1:**
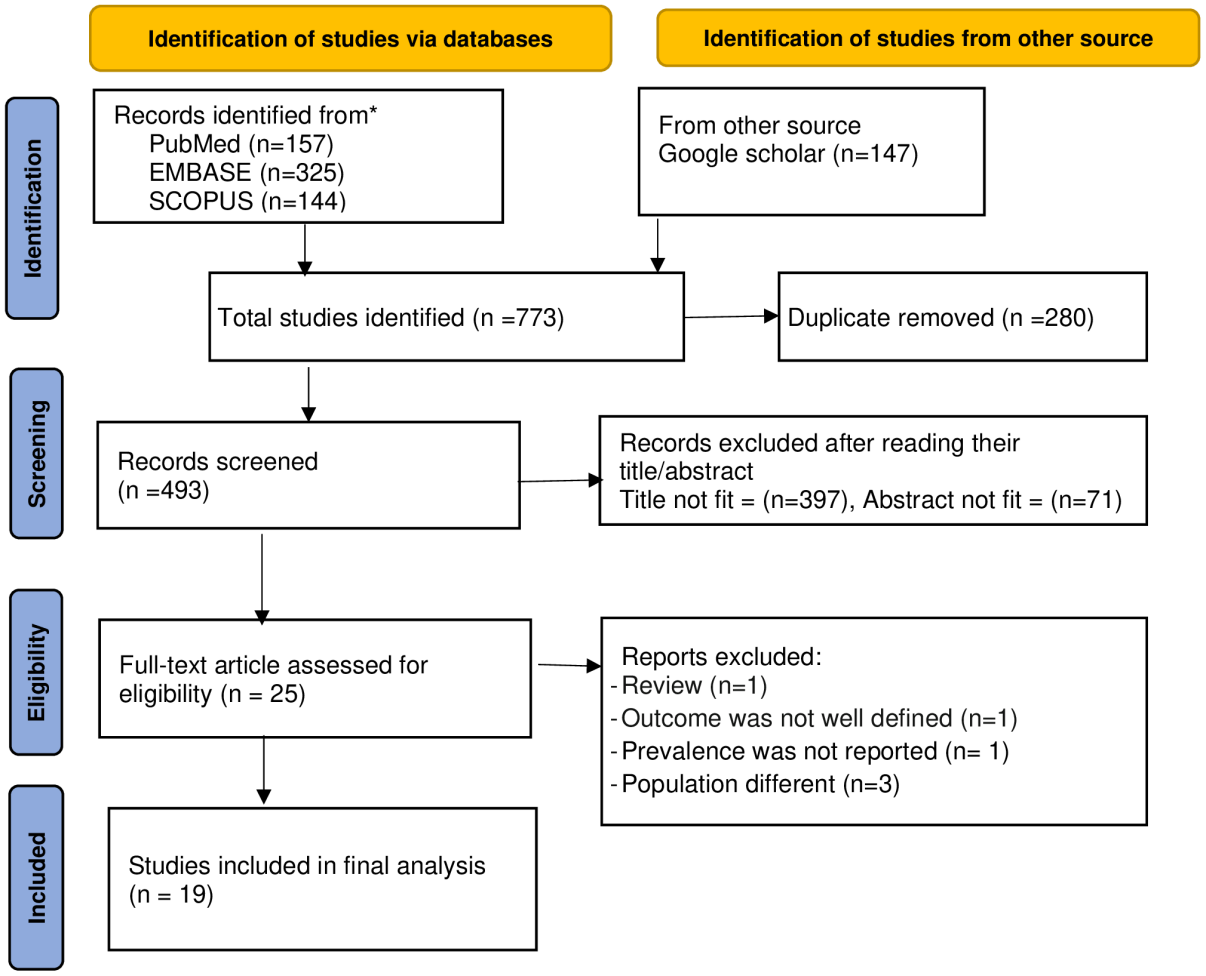
PRISMA 2020 flow diagram for systematic reviews and meta-analyses of chronic kidney disease and its associated factors among DM patients in Ethiopia, 2024.

For this systematic review and meta-analysis, a total of 773 articles were found using various electronic databases. After the initial exclusion of duplicates (280), 493 articles were screened based on the title and abstract. After screening the titles and abstracts, 25 patients were eligible for full-text review. Finally, 19 articles fulfilled the inclusion criteria and were included in the systematic review and meta-analysis (Fig 1).

### Characteristics of the included studies

In this study, a total of 19 studies with 6,489 participants were included and used to estimate the pooled prevalence of CKD among diabetic patients in Ethiopia. In terms of study design, the majority (74%) of the studies were conducted with a cross-sectional study design, and 3,410 of the subjects who participated in the studies were males.

The included studies had substantial variation in sample size, ranging from 199 from the Tigray region [14] to 626 from the SNNP region [12]. The prevalence of CKD in patients with DM was obtained from various regions in Ethiopia; eight studies were from Amhara [10,26–32], four studies were from Oromia [33–36], four were from Addis Ababa [15,37–39], one was from the SNNPR [12], one was from the Tigray [14], and one was from the Harari region [9]. Furthermore, we evaluated the quality of the included studies using JBI critical appraisal checklists. Nearly 90% of the studies were categorized as high quality, indicating a low risk of bias and strong reliability of the findings. (Table 1).

**Table 1:** Baseline characteristics of the included studies for the prevalence of chronic kidney disease among diabetes mellitus patients in Ethiopia, 2024.

Id	Author	Year	Region	Study design	Number of participants	CKD prevalence %	Quality score
1	Fiseha et al. [36]	2014	Oromia	Cross-sectional	214	23.8	Low risk
2	Geletu, A. [37]	2018	Addis Ababa	Retrospective follow-up	435	14.2	Low risk
3	Damtie et al. [31]	2018	Amhara	cross-sectional	229	21.8	Moderate risk
4	Kumela Goro et al. [35]	2019	Oromia	Cross-sectional	208	26.1	Low risk
5	Asfeha et al. [14]	2020	Tigray	Cross-sectional	199	21.9	Low risk
6	Alemu et al. [28]	2020	Amhara	Cross-sectional	272	17.3	Low risk
7	Taderegew [30]	2020	Amhara	Cross-sectional	422	24.4	Low risk
8	Fiseha and Tamir [32]	2020	Amhara	Cross-sectional	323	13.0	Low risk
9	Tamru et al. [39]	2020	Addis Ababa	Retrospective follow-up	346	19.6	Low risk
10	Amanuel Berhanu [38]	2021	Addis Ababa	Cross-sectional	308	23.1	Low risk
11	Debele et al. [34]	2021	Oromia	Retrospective follow-up	405	15.5	Low risk
12	Hailu et al. [33]	2022	Oromia	Cross-sectional	308	20.5	Low risk
13	Tesfe et al. [26]	2022	Amhara	Cross-sectional	329	16.7	Moderate risk
14	Ahmed et al. [29]	2022	Amhara	Retrospective follow-up	415	10.8	Low risk
15	Abdulkadr et al. [15]	2022	Addis Ababa	Cross-sectional	362	14.6	Low risk
16	Cheru et al. [9]	2023	Harari	Retrospective follow-up	494	10.3	Low risk
17	Mulu et al. [27]	2023	Amhara	Cross-sectional	327	15.9	Low risk
18	Israel et al. [12]	2024	SNNP	Cross-sectional	626	2.7.0	Low risk
19	Adem et al. [10]	2024	Amhara	Cross-sectional	267	25.4	Low risk

CKD: chronic kidney disease, SNNP: southern nation-nationality people.

### Pooled prevalence of chronic kidney disease in Ethiopia

**Fig 2:**
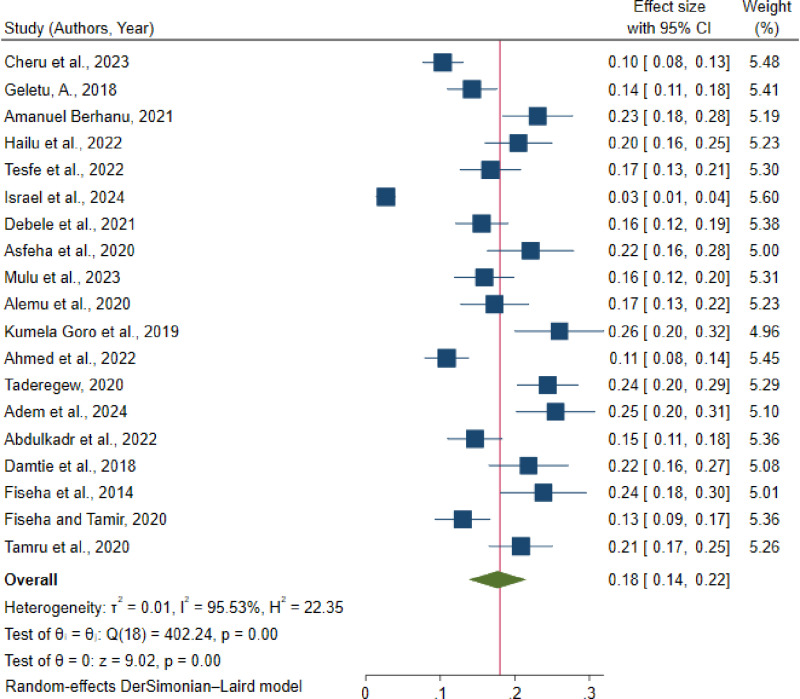
Forest plot of the pooled prevalence of chronic kidney disease among diabetic mellitus patients in Ethiopia, 2024.

According to the random effects model, the overall pooled prevalence of CKD in Ethiopia was 18% (95% CI: 14, 22%). Significant heterogeneity was observed among the studies (I^2^ = 95.5, p-value < 0.001). The prevalence of CKD ranged from the lowest 3% reported from SNNP [12] to the highest prevalence of 25% reported from the Oromia region [35] (Fig 2).

### Publication bias

The assessment of publication bias included a visual inspection of funnel plots, and Egger’s test was performed. The funnel plot demonstrated noticeable asymmetry, suggesting potential publication bias (S1 Fig). Additionally, Egger’s regression test was employed to objectively confirm publication bias, which resulted in a statistically significant p-value (p = 0.001), indicating the presence of publication bias. This finding suggested that smaller studies with no significant results might be underrepresented in our analysis.

To address publication bias further, we applied the trim-and-fill method. After we adjusted for these missing studies, the pooled prevalence of CKD among DM patients was revised from 18.0% (95% CI: 14, 21%) to 17.7% (95% CI: 13, 21%), the adjusted effect size was not significantly different from the original estimate. Therefore, publication bias is not a concern in this systematic review and meta-analysis. The adjusted estimate provides a more balanced view, accounting for the potential overrepresentation of significant findings (S2 Fig).

### Source of heterogeneity and handling

The pooled estimate of the random effect model revealed significant heterogeneity. As a result, subgroup analysis, sensitivity analyses, and meta-regression were performed to identify the source of heterogeneity.

### Subgroup analyses

Subgroup analysis based on the study design, type of DM, and sample size was performed.

**Fig 3:**
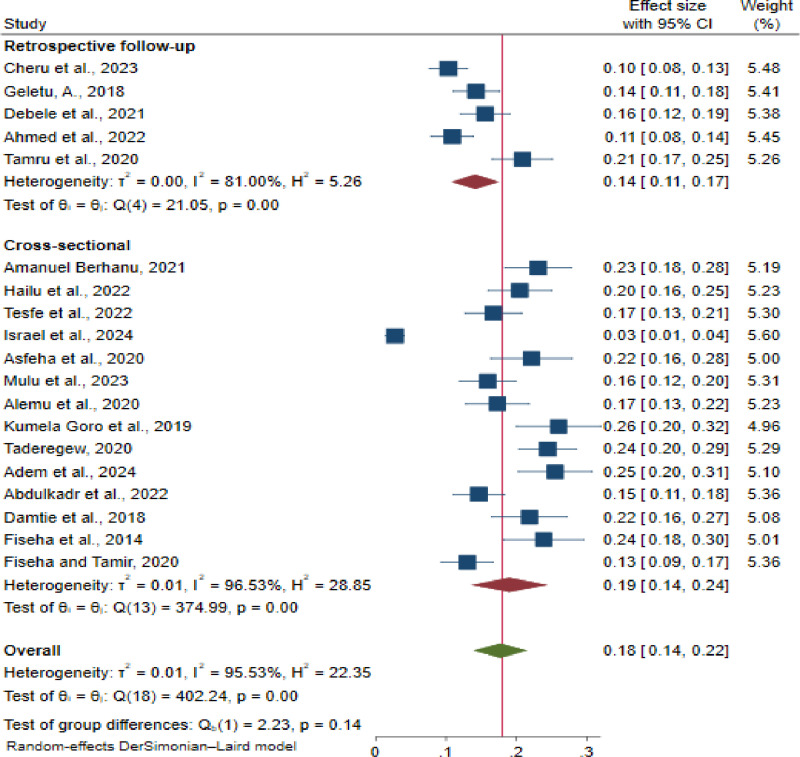
Subgroup analysis of chronic kidney disease prevalence among diabetes mellitus patients, based on study designs of the primary studies in Ethiopia, 2024.

The pooled prevalence of CKD was estimated to be 14% for studies performed via retrospective follow-up studies (95% CI: 11.0, 17.0; I^2^ = 81.0%, p-value < 0.001). This was lower than the pooled prevalence for studies done by cross-sectional studies, which was 19% (95% CI: 14.0, 24.0; I^2^ = 96.53%; p-value < 0.001) (Fig 3).

**Fig 4:**
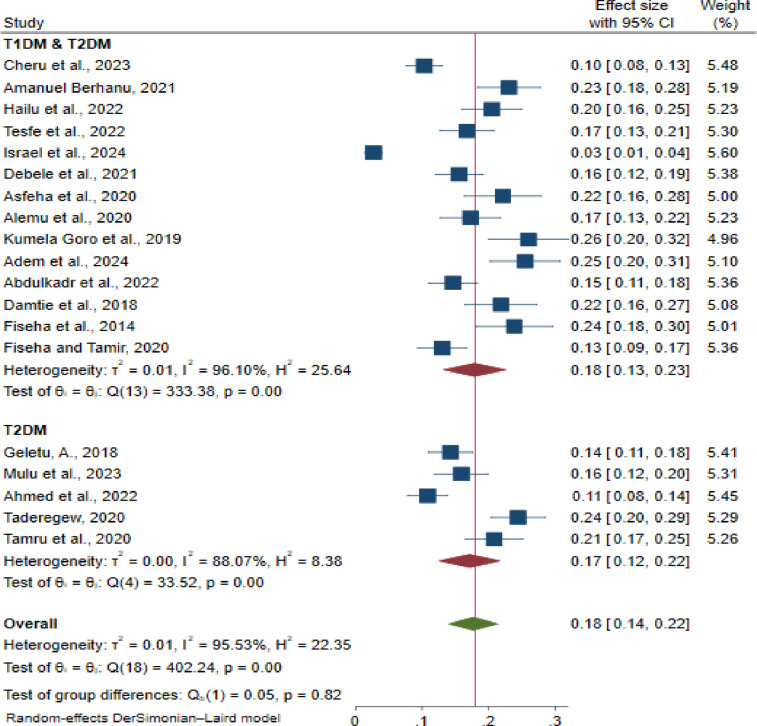
Subgroup analysis of the prevalence of chronic kidney disease based on type of diabetes mellitus in primary studies in Ethiopia, 2024.

To investigate the differences in CKD prevalence among diabetic subtypes, we conducted a subgroup analysis. The analysis revealed that the pooled prevalence of CKD in patients with T2DM was 17% (95% CI: 12.0, 22.0; I² = 88.7%, p < 0.001). This prevalence is lower than the pooled prevalence observed for both T1DM and T2DM, which was 18% (95% CI: 13.0, 23.0; I² = 96.1%, p < 0.001) (Fig 4).

**Fig 5:**
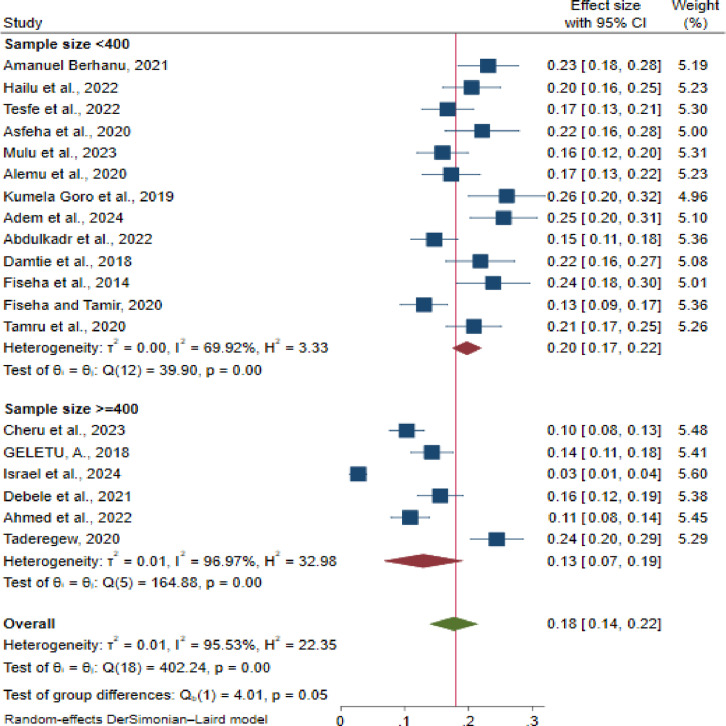
Subgroup analysis of the prevalence of chronic kidney disease among patients with diabetes mellitus, based on the sample sizes of the primary studies in Ethiopia, 2024.

Furthermore, the subgroup analysis based on the study sample size revealed significant variations in CKD prevalence. Studies with a sample size of lower than 400 participants reported a higher CKD prevalence of 20% (95% CI: 17, 22; I² = 69.92%, p < 0.001) compared to those with 400 or more study participants, which showed a CKD prevalence of 13% (95% CI: 7, 19; I² = 96.97%, p < 0.001) (Fig 5).

### Sensitivity analysis

A sensitivity analysis was performed using a random-effects model to determine the impact of individual studies on overall prevalence estimates. The findings showed that no single study had a significant impact on the pooled estimate of chronic kidney disease, as the overall estimate remained consistent when each study was omitted sequentially from the analysis. See (S3 Fig).

### Meta-regression

To identify the source of heterogeneity in the main studies that were included, we used univariate meta-regression. Using the sample size, response rates, study participant mean age, and year of publication as covariates revealed no significant associations. (Table 2).

**Table 2:** Univariate meta-regression analysis results for the pooled prevalence of chronic kidney disease among diabetes mellitus patients in Ethiopia, 2024.

Covariates	Coefficients (95% CI)	Standard error	P-value
Mean age	0.004 (−0.045, 0.007)	0.002	0.750
Publication year	0.039 (−0.017, 0.236)	0.039	0.227
Sample size	0.279 (−0.656, 2.215)	0.987	0.777
Response rates	−0.001 (−0.021, 0.018)	0.010	0.898

### Factors associated with chronic kidney disease among DM patients

**Fig 6:**
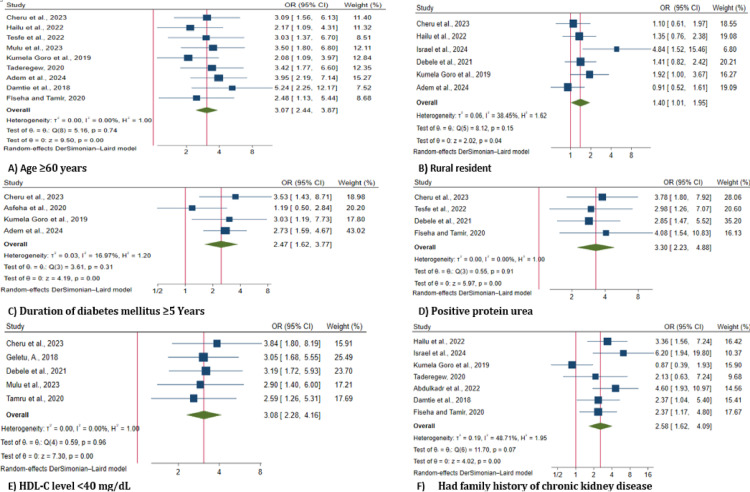
Forest plot for factors associated with chronic kidney disease among diabetes mellitus patients in Ethiopia, 2024.

Separate random effects pooled estimate analysis was conducted on the extracted factors, such as age, sex, residence, type of diabetes, duration of diabetes, presence of protein urea, HDL level, family history of CKD, and income, to identify factors associated with the prevalence of CKD in Ethiopia. Therefore, factors such as age 60 years or older, rural residence, duration of diabetes, presence of protein urea, HDL-C level, and family history of CKD were found to be significantly associated to the prevalence of CKD among DM patients in Ethiopia (Fig 6).

A pooled meta-analysis revealed that DM patients aged ≥ 60 years were 3.0 times more likely to have CKD compared to those younger than 60 years (OR = 3.07 (95% CI: 2.44, 3.87); I^2^ = 0.01%). DM patients who were from rural residents had a 1.4-fold greater risk for CKD than their counterparts (OR = 1.40 (95% CI: 1.01, 1.95), I^2^ = 38.4%). Similarly, compared with patients with less than five years of DM, those with more than five years of DM were 2.4 times more likely to develop CKD (OR = 2.47 (95% CI: 1.62, 3.77); I^2^ = 17.0%). Patients with positive proteinuria were 3.3 times more likely to develop CKD than those with negative proteinuria (OR = 3.30 (95% CI: 2.23, 4.88); I^2^ = 0.01%). An HDL-C level of < 40 mg/dL was significantly associated with an increased risk of CKD (OR: 3.08; 95% CI: 2.28, 4.16; I² = 0.01%) compared to an HDL-C level of ≥ 40 mg/dL. Concerning family history of CKD, DM patients who had a family history of CKD were 2.5 times more likely to have CKD than those who had no family history (OR = 2.58 (95% CI: 1.62, 4.09), I^2^ = 48.7%) (Table 3).

**Table 3:** Summary of the pooled effects of factors associated with the prevalence of chronic kidney disease among diabetes mellitus patients in Ethiopia, 2024.

Variable	Category	OR (95% CI)	Heterogeneity (I^2^, P-value)	Egger’s P-value	Total studies	Sample size
Age	< 60 year	1	1			
	≥60 year	3.07 (2.44, 3.87)*	0.01% (0.741)	0.270	9	2,907
Sex	Female	1	1			
	Male	1.26 (0.95, 1.67)	51.81% (0.035)	0.334	9	3,069
Residence	Urban	1	1			
	Rural	1.40 (1.01, 1.95)*	38.41% (0.150)	0.042	6	2,308
Types of diabetes mellitus	Type I DM	1	1			
	Type II DM	1.17 (0.63, 2.18)	48.30% (0.085)	0.003	6	2,123
Duration of DM	<5 Years	1	1			
	≥5 Years	2.47 (1.62, 3.77)*	17.01% (0.306)	0.835	4	1,168
Presence of protein urea	Negative	1	1			
	Positive	3.30 (2.23, 4.88)*	0.01% (0.907)	0.437	4	1,551
HDL-C level	≥40 mg/dL	1	1			
	<40 mg/dL	3.08 (2.28, 4.16)*	0.01% (0.957)	0.531	5	1,661
Family History of CKD	No	1	1			
	Yes	2.58 (1.62, 4.09)*	48.70% (0.069)	0.502	7	2,478
Income	< 1500 Birr	1	1			
	≥ 1500 Birr	0.87 (0.18, 4.27)	83.50% (0.014)	0.343	2	466

*Statistically significant at 5% level, OR: Odds Ratio, CI: Confidence Interval, I^2^: I-squared.

## Discussions

CKD is a significant health concern among patients with DM. It affects millions of individuals globally and contributes to increased morbidity and mortality rates. This systematic review and meta-analysis aimed to estimate the pooled prevalence of CKD among DM patients in Ethiopia and compare it with findings from other regions.

Our analysis included 6,489 participants from 19 eligible studies. The overall pooled prevalence of CKD among DM patients in Ethiopia was 18% (95% CI: 14%, 22%). This finding is consistent with those of studies conducted in South Africa [40] and Bangladesh [41], which reported that the prevalence of CKD was 17% and 21%, respectively. However, this study result is higher than the prevalence reported in a systematic review in Sub-Saharan countries, which was 13.9% [42]. On the other hand, this prevalence is lower than reported in Nigeria [43] and Middle East countries [44], which was 28.96%. The high prevalence of CKD among DM patients in Ethiopia highlights significant gaps in the management of diabetes.

The variability in pooled estimates could be attributed to differences in healthcare infrastructure, access to medications, and the timing of studies, which can significantly impact pooled prevalence rates. Additionally, the timing of studies can affect prevalence estimates. Variations in healthcare policies, advancements in treatment options, and public health interventions over time can influence the management and prevention of CKD in diabetic patients. Studies conducted at different times might reflect improvements or deterioration in healthcare practices and disease management, contributing to differences in reported prevalence rates.

Additionally, this study identified several associated factors contributing to NCD prevalence among DM patients in Ethiopia. Key factors, such as age 60 years or older, rural residence, longer duration of diabetes, presence of protein urea, lower HDL-C level, and family history of CKD, were associated with the prevalence of CKD among DM patients. Understanding these factors is essential for developing targeted interventions to improve CKD prevention and management in this population.

This study revealed that a DM patient age of 60 years and older was associated with a higher prevalence of CKD. This finding is supported by studies conducted in Ghana that revealed that the prevalence of CKD increased with increasing age [45]. Another global study on the epidemiology of chronic kidney disease showed that the prevalence of CKD increases linearly with age [1]. Naturally, the kidney’s capacity to function gradually decreases as age increases [46]. In addition, conditions such as cardiovascular disease and hypertension, which are risk factors for CKD, are more common in older adults [47]. Moreover, older people might be exposed to different CKD risk factors, such as certain drugs and toxins, for a longer time. All of these factors may collectively contribute to the higher prevalence of CKD in older adults [48,49].

DM patients from rural areas were 1.4 times more likely to develop CKD than those from urban areas. This result is supported by a study in China in which the risk of CKD was found to be greater in rural areas than in urban areas [50]. Another study from Taiwan indicated that patients living in rural areas had higher rates of diabetes-related complications than patients living in urban areas [51]. This could be because people living in rural areas often face a greater risk of various health issues because of limited access to healthcare services, lower socioeconomic status, and fewer health education resources.

Similarly, DM patients who had durations greater than five years were 2.47 times more likely to develop CKD than those who had durations less than five years. This result, supported by a study in Malaysia, demonstrated that the risk of CKD dramatically increases with the duration of diabetes [52]. This increased risk results from extended exposure to hyperglycemia, which may deteriorate the microvascular structure of the kidneys over time, increasing the risk of CKD. Therefore, to reduce the risk of developing CKD in diabetic patients, this correlation highlights the importance of early diagnosis, efficient management, and routine kidney function monitoring.

DM patients with positive proteinuria had a higher risk of developing CKD than their counterparts. This result is consistent with research conducted in India [53], which showed that DM patients experienced a 1.7-fold increase in incident CKD for every doubling of the albumin-to-creatinine ratio. Another study in Japan [54] revealed that patients with normal-range proteinuria had a significantly lower risk of developing CKD than those with higher-range proteinuria. Recent data indicate that proteinuria itself has a direct toxic effect on renal tissues [55]. The filtered proteins can cause inflammation and injury to kidney tubular cells, leading to a cascade of events that result in fibrosis and further loss of kidney function.

In this study, we found that lower levels of HDL-C (<40 mg/dl) were associated with a higher risk of CKD. This result was supported by the findings of another study in China [56], which showed that high levels of HDL-C increase the risk of diabetic kidney disease, also study in Italy [57] revealed that higher HDL-C levels were associated with lower incidence of CKD in DM patients. This is because lower HDL-C levels promote atherosclerosis, increase inflammation and oxidative stress cause endothelial dysfunction [58], and often coexist with other metabolic risk factors, leading to a multifaceted impact on renal health.

Regarding the family history of CKD, DM patients who had a family history of CKD were 2.5 times more likely to develop CKD than those who did not. Additionally, a study in China [59] revealed that individuals with a family history of CKD had a 1.4-fold increased risk of developing CKD compared with those without a family history of CKD. This could be genetic predispositions, along with similar environmental and lifestyle factors such as dietary habits, physical activity levels, and exposure to environmental factors, may significantly contribute to the development of CKD in this population.

This study has some strengths and limitations. The study’s strengths include providing valuable insights by synthesizing data from various regions of Ethiopia and offering a comprehensive and robust estimate of the pooled prevalence of CKD among DM patients. It also identifies key sociodemographic and clinical factors, which can inform targeted interventions for at-risk populations. Furthermore, the use of rigorous statistical methods such as forest plots, subgroup analysis, sensitivity analysis, and meta-regression, were used to enhance the validity and reliability of the findings. However, some limitations were observed. The first one is the high heterogeneity across studies, indicating significant variability in effect sizes due to differences in study designs, population characteristics, and sample sizes. To address this, a random-effects model and subgroup analyses were employed. Additionally, the exclusion of unpublished studies might limit the breadth of available data, potentially introducing publication bias. Moreover, the lack of data on certain variables, such as income and behavioral factors, is another limitation that could affect the comprehensiveness of the study’s findings.

## Conclusion

The prevalence of CKD in Ethiopia was high, affecting nearly two in five individuals with diabetes. In addition, factors such as older age, rural residence, longer DM duration, positive proteinuria, lower HDL levels, and a family history of CKD were significantly associated with CKD prevalence. Therefore, targeted public health interventions, such as screening, education, and awareness programs, are highly recommended to mitigate this problem.

## Supporting information

S1 Fig Funnel plot for assessing publication bias in the prevalence of chronic kidney disease among diabetic mellitus patients in Ethiopia, 2024. (TIF)

S2 Fig Trim-and-fill analysis on the pooled estimates of chronic kidney disease among diabetic mellitus patients in Ethiopia, 2024. (TIF)

S3 Fig Sensitivity analysis of studies included in the pooled estimates of chronic kidney disease prevalence among diabetes mellitus patients in Ethiopia, 2024. (TIF)

S1 File Data file. (XLSX)

S2 File **S1 Table:** Search Strategy in Included Databases, for chronic kidney disease among diabetic mellitus patients in Ethiopia, 2024. **S2 Table.** Quality assessment for cross-sectional studies. **S3 Table.** Quality assessment for retrospective cohort study.
(DOCX)
