# Supplementary figures and images for: Prevalence and associated factors of chronic kidney disease among diabetes mellitus patients in Ethiopia: A systematic review and meta-analysis
Source: PLoS One. 2025 Mar 5;20(3):e0315529. doi: 10.1371/journal.pone.0315529 (PMC11882046; doi:10.1371/journal.pone.0315529)

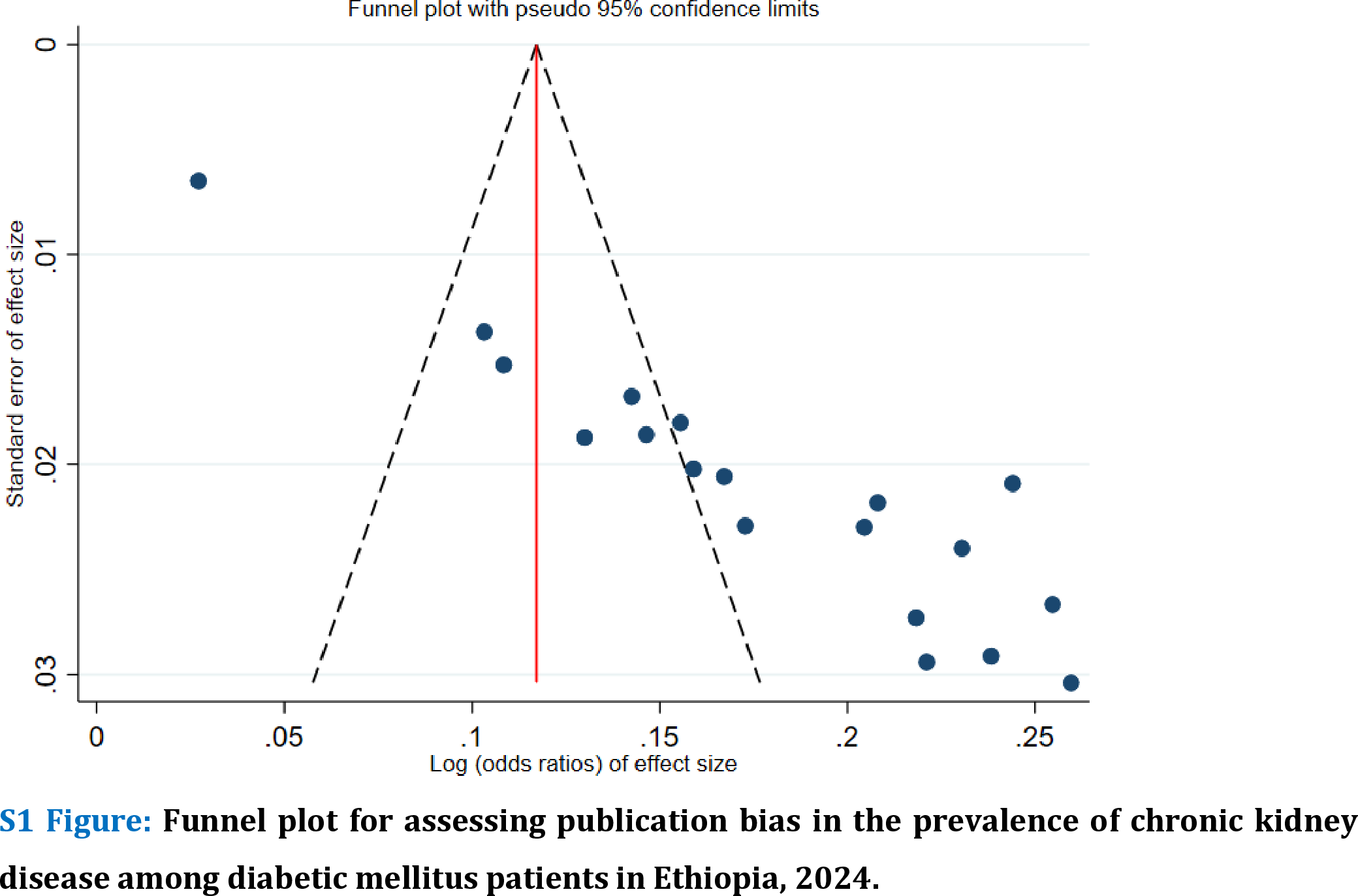

Supplement: S1 Fig — (TIF) [file pone.0315529.s001.tif]

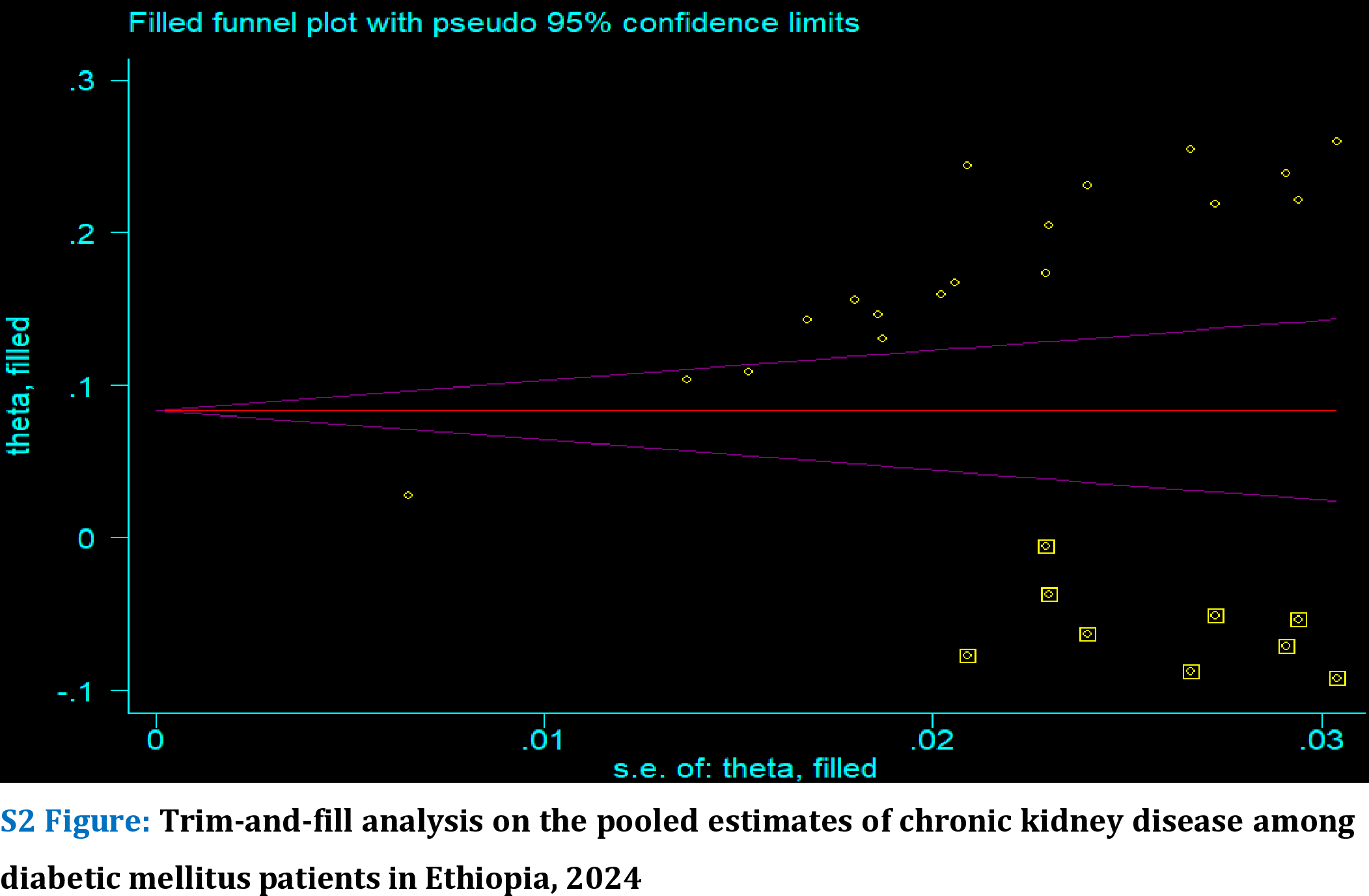

Supplement: S2 Fig — (TIF) [file pone.0315529.s002.tif]

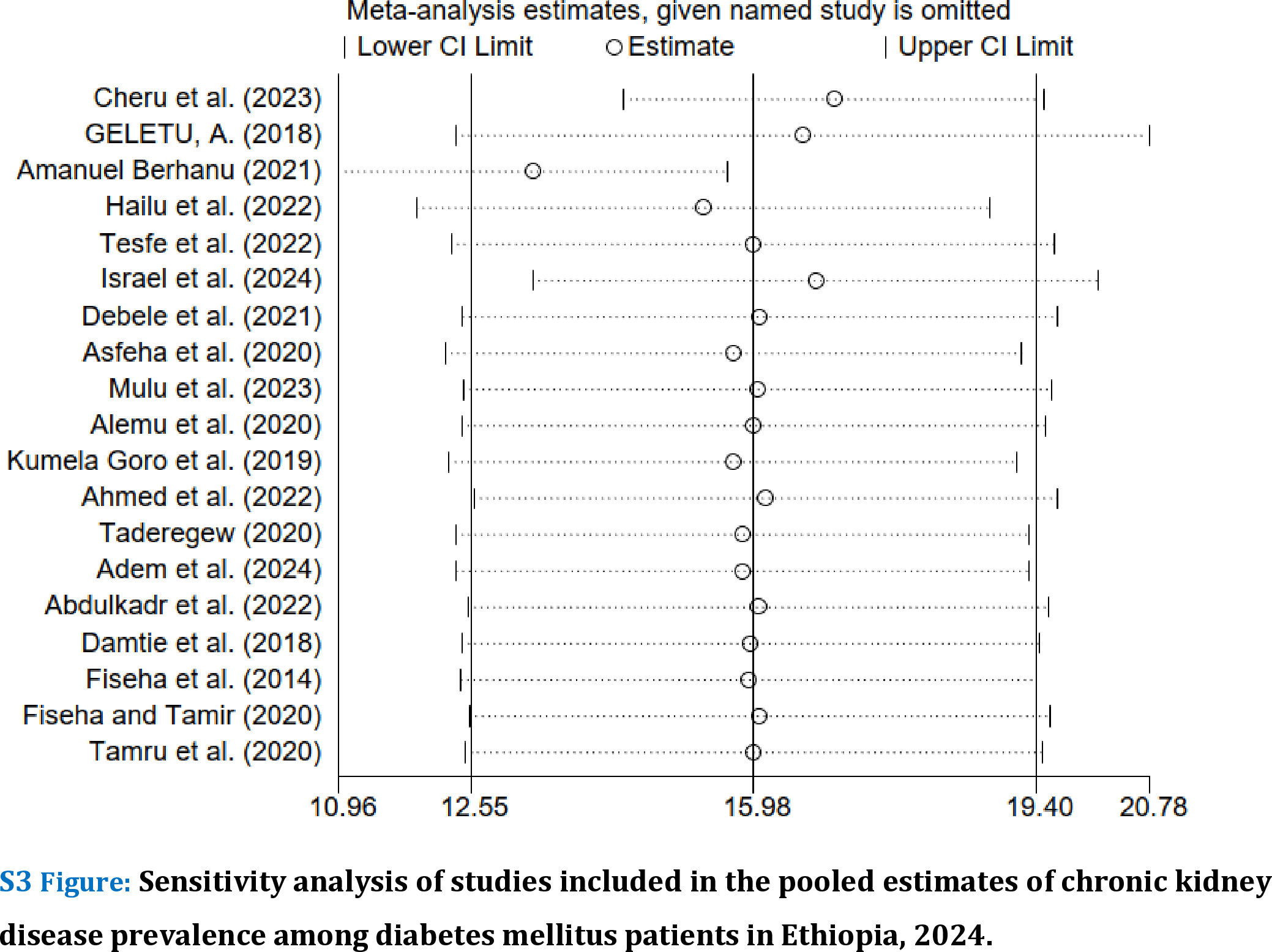

Supplement: S3 Fig — (TIF) [file pone.0315529.s003.tif]
