# Supplementary material for: Prevalence and associated factors of chronic kidney disease among diabetes mellitus patients in Ethiopia: A systematic review and meta-analysis
Source: PLoS One. 2025 Mar 5;20(3):e0315529. doi: 10.1371/journal.pone.0315529 (PMC11882046; doi:10.1371/journal.pone.0315529)
Supplement: S2 File — S1 Table: Search Strategy in Included Databases, for chronic kidney disease among diabetic mellitus patients in Ethiopia, 2024. S2 Table. Quality assessment for cross-sectional studies. S3 Table. Quality assessment for retrospective cohort study. (DOCX) [file pone.0315529.s005.docx]

**Supplementary 1 Table: Search Strategy in Included Databases, for chronic kidney disease among diabetic mellitus patients in Ethiopia, 2024.**

| **Data bases** | **Search strategy** |
| --- | --- |
| PubMed | ((Diabetes OR "diabetes mellitus" OR "Diabetes Mellitus"[Mesh] OR "type 1 diabetes" OR "type 1 diabetes mellitus" OR T1DM OR "type 2 diabetes" OR "type 2 diabetes mellitus" OR T2DM) AND ("Chronic kidney disease" OR "diabetic nephropathy" OR "Diabetic Nephropathies"[Mesh] OR "chronic renal failure" OR "renal impairment" OR proteinuria OR "end-stage kidney disease" OR "end-stage renal disease" OR "renal insufficiency") AND (Ethiopia OR Tigray OR Afar OR Amhara OR "Benishangul-Gumuz" OR Gambela OR Harari OR Oromia OR Sidama OR Somali OR "Southern Nations Nationalities and Peoples" OR "Addis Ababa" OR "Dire Dawa")) Searched up to 2024/8/15. =157 articles |
| Embase | ('diabetes'/exp OR diabetes OR 'diabetes mellitus'/exp OR 'diabetes mellitus' OR 'type 1 diabetes'/exp OR 'type 1 diabetes' OR 'type 1 diabetes mellitus'/exp OR 'type 1 diabetes mellitus' OR 't1dm'/exp OR t1dm OR 'type 2 diabetes'/exp OR 'type 2 diabetes' OR 'type 2 diabetes mellitus'/exp OR 'type 2 diabetes mellitus' OR 't2dm'/exp OR t2dm) AND ('chronic kidney disease'/exp OR 'chronic kidney disease' OR 'diabetic nephropathy'/exp OR 'diabetic nephropathy' OR 'diabetic nephropathies'/exp OR 'diabetic nephropathies' OR 'chronic renal failure'/exp OR 'chronic renal failure' OR 'renal impairment'/exp OR 'renal impairment' OR 'proteinuria'/exp OR proteinuria OR 'end-stage kidney disease'/exp OR 'end-stage kidney disease' OR 'end-stage renal disease'/exp OR 'end-stage renal disease' OR 'renal insufficiency'/exp OR 'renal insufficiency') AND ('ethiopia'/exp OR ethiopia OR tigray OR afar:ab,ti OR amhara OR 'benishangul-gumuz' OR gambela OR harari OR oromia OR sidama OR somali OR 'southern nations nationalities and peoples' OR 'addis ababa' OR 'dire dawa':ab,ti) AND [english]/lim **=** 325 articles |
| Scopus | TITLE-ABS-KEY (diabetes) OR TITLE-ABS-KEY ("diabetes mellitus") OR TITLE-ABS-KEY ("Diabetes Mellitus") OR TITLE-ABS-KEY ("type 1 diabetes") OR TITLE-ABS-KEY ("type 1 diabetes mellitus" ) OR TITLE-ABS-KEY ( t1dm ) OR TITLE-ABS-KEY ( "type 2 diabetes" ) OR TITLE-ABS-KEY ( "type 2 diabetes mellitus" ) OR TITLE-ABS-KEY ( t2dm ) AND TITLE-ABS-KEY ( "Chronic kidney disease" ) OR TITLE-ABS-KEY ("diabetic nephropathy" ) OR TITLE-ABS-KEY ( "Diabetic Nephropathies" ) OR TITLE-ABS-KEY  ("chronic renal failure") OR TITLE-ABS-KEY ("renal impairment") OR TITLE-ABS-KEY  (proteinuria) OR TITLE-ABS-KEY ("end-stage kidney disease") OR TITLE-ABS-KEY  ("end-stage renal disease") OR TITLE-ABS-KEY ("renal insufficiency") AND TITLE-ABS-KEY (ethiopia) OR TITLE-ABS-KEY (tigray) OR TITLE-ABS-KEY (afar) OR TITLE-ABS-KEY (amhara ) OR TITLE-ABS-KEY ( "Benishangul-Gumuz" ) OR TITLE-ABS-KEY  (gambela) OR TITLE-ABS-KEY (harari) OR TITLE-ABS-KEY (Oromia) OR TITLE-ABS-KEY (sidama) OR TITLE-ABS-KEY (somali) OR TITLE-ABS-KEY ("Southern Nations Nationalities and Peoples" ) OR TITLE-ABS-KEY ( "Addis Ababa" ) OR TITLE-ABS-KEY  ("Dire Dawa") AND (LIMIT-TO (LANGUAGE, "English")) AND (LIMIT-TO  ( EXACTKEYWORD , "Article" ) ) **=**144 articles |
| Google scholar | **With all of word:** prevalence of chronic Kidney disease among diabetes patient in Ethiopia  **with the exact phrases:** chronic Kidney disease prevalence OR incidence  **At least one of those word**: Ethiopia  Searched **=**147 articles |

Legend: AB: abstract, MH: MesH term, TI: title, TW: text word

**Supplementary 2 Table: Risk of bias assessment for cross-sectional studies.**

| **Id** | **Author** | **C1** | **C2** | **C3** | **C4** | **C5** | **C6** | **C7** | **C8** | #Total score | **Quality score** |
| --- | --- | --- | --- | --- | --- | --- | --- | --- | --- | --- | --- |
| 1 | Fiseha et al. 2014 | Y | Y | Y | Y | N/A | N/A | Y | Y | 6 | Low risk |
| 2 | [Damtie et al.](#RANGE!_ENREF_33)  2018 | Y | N | U | Y | N/A | N/A | U | Y | 4 | Moderate risk |
| 3 | Kumela Goro et al. 2019 | Y | Y | Y | U | N/A | N/A | Y | Y | 5.5 | Low risk |
| 4 | [Asfeha et al.](#RANGE!_ENREF_15)  2020 | Y | Y | U | Y | N/A | N/A | Y | Y | 5 | Low risk |
| 5 | Alemu et al. 2020 | Y | Y | Y | N | N/A | N/A | Y | Y | 5 | Low risk |
| 6 | [Taderegew](#RANGE!_ENREF_31)  2020 | Y | Y | Y | Y | N/A | N/A | Y | Y | 6 | Low risk |
| 7 | Fiseha and Tamir 2020 | Y | Y | Y | U | N/A | N/A | Y | Y | 5 | Low risk |
| 8 | Amanuel Berhanu 2021 | Y | Y | Y | Y | N/A | N/A | Y | Y | 6 | Low risk |
| 9 | Hailu et al. 2022 | Y | Y | Y | Y | N/A | N/A | Y | Y | 6 | Low risk |
| 10 | Tesfe et al. 2022 | U | Y | Y | N | N/A | N/A | Y | U | 4 | Moderate risk |
| 11 | Abdulkadr et al. 2022 | Y | Y | Y | Y | N/A | N/A | Y | Y | 6 | Low risk |
| 12 | Mulu et al. 2023 | Y | Y | U | Y | N/A | N/A | Y | Y | 5 | Low risk |
| 13 | Israel et al. 2024 | Y | Y | Y | Y | N/A | N/A | Y | Y | 6 | Low risk |
| 14 | Adem et al. 2024 | Y | Y | Y | Y | N/A | N/A | Y | Y | 6 | Low risk |

Y = Yes; N = No; U – Unclear; N/A - Not/Applicable

C1: Were the criteria for inclusion in the sample clearly defined? C2: Were the study subjects and the setting described in detail? C3: Was the exposure measured in a valid and reliable way?

C4: Were objective, standard criteria used for measurement of the condition? C5: Were confounding factors identified? C6: Were strategies to deal with confounding factors stated?

C7: Were the outcomes measured in a valid and reliable way? C8: Was appropriate statistical analysis used?

Summarizing Scores

“Yes” was given a value of 1, the score “No” was given a value of 0, the score “Unclear” was given a value of 0.5, and “Not applicable was denoted as N/A.

Interpreting the Scores

Low risk of bias (High quality) was nominated if the study scored 6 and above, moderate risk of bias (Good quality) if the study scored between 5 and 4, and High risk of bias (lower quality) for studies scored below 3.

**Supplementary 3 Table: Risk of bias assessment for retrospective cohort study**

| **Id** | **Author** | **C1** | **C2** | **C3** | **C4** | **C5** | **C6** | **C7** | **C8** | **C9** | **C10** | **C11** | **#Total score** | **Quality score** |
| --- | --- | --- | --- | --- | --- | --- | --- | --- | --- | --- | --- | --- | --- | --- |
| 1 | [Geletu, A.](#RANGE!_ENREF_39)  2018 | Y | U | Y | N/A | N/A | Y | Y | Y | Y | U | Y | 8 | Low risk |
| 2 | [Tamru et al.](#RANGE!_ENREF_41)  2020 | Y | U | Y | N/A | N/A | Y | Y | Y | Y | Y | Y | 9 | Low risk |
| 3 | Debele et al. 2021 | Y | Y | Y | N/A | N/A | Y | Y | Y | Y | Y | Y | 9 | Low risk |
| 4 | [Ahmed et al.](#RANGE!_ENREF_30)  2022 | Y | U | U | N/A | N/A | Y | Y | Y | Y | N | Y | 8 | Low risk |
| 5 | Cheru et al. 2023 | Y | Y | Y | N/A | N/A | Y | Y | Y | Y | Y | Y | 9 | Low risk |

Y = Yes; N = No; U – Unclear; N/A - Not/Applicable

C1: Were the two groups similar and recruited from the same population? C2: Were the exposures measured similarly to assign people to both exposed and unexposed groups? C3: Was the exposure measured in a valid and reliable way? C4: Were confounding factors identified? C5: Were strategies to deal with confounding factors stated? C6: Were the groups/participants free of the outcome at the start of the study (or at the moment of exposure)? C7: Were the outcomes measured in a valid and reliable way? C8: Was the follow up time reported and sufficient to be long enough for outcomes to occur? C9: Was follow up complete, and if not, were the reasons to loss to follow up described and explored? C10: Were strategies to address incomplete follow up utilized? C11: Was appropriate statistical analysis used?

Summarizing Scores

“Yes” was given a value of 1, the score “No” was given a value of 0, the score “Unclear” was given a value of 0.5, and “Not applicable was denoted as N/A.

Interpreting the Scores

Low risk of bias (High quality) was nominated if the study scored 8 and above, moderate risk of bias (Good quality) if the study scored between 7 and 5.5, and High risk of bias (lower quality) for studies scored below 5.


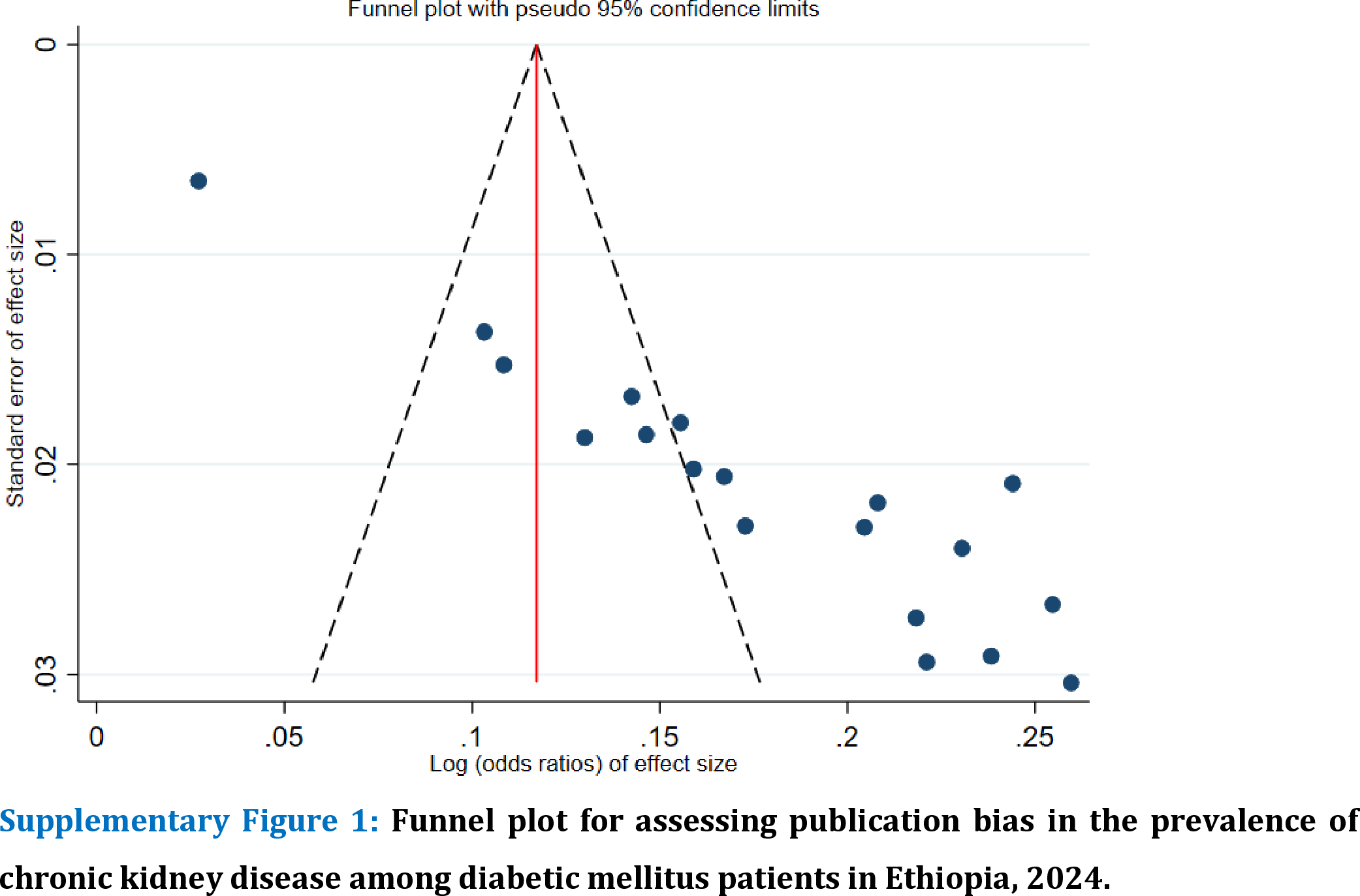


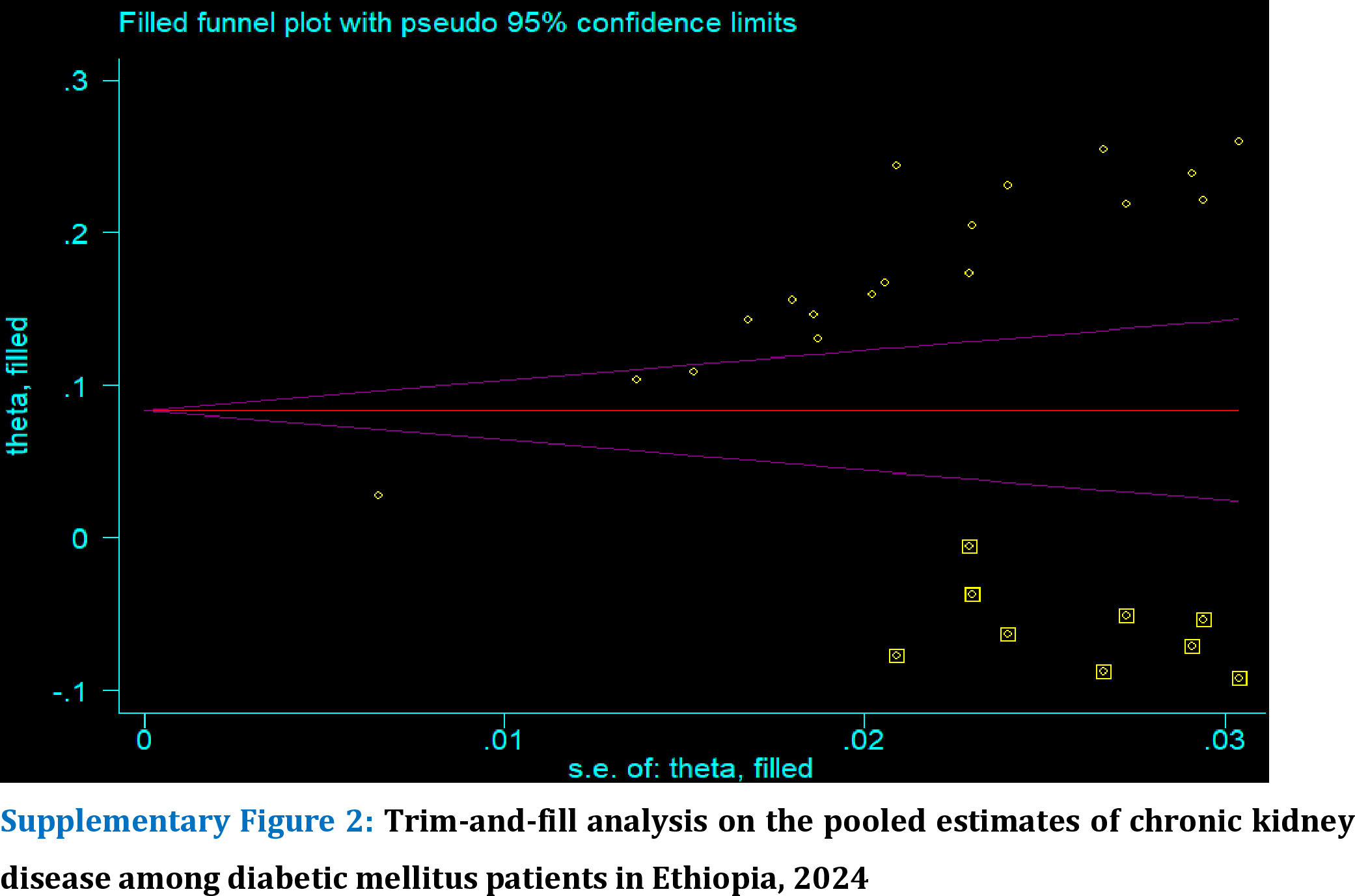


**
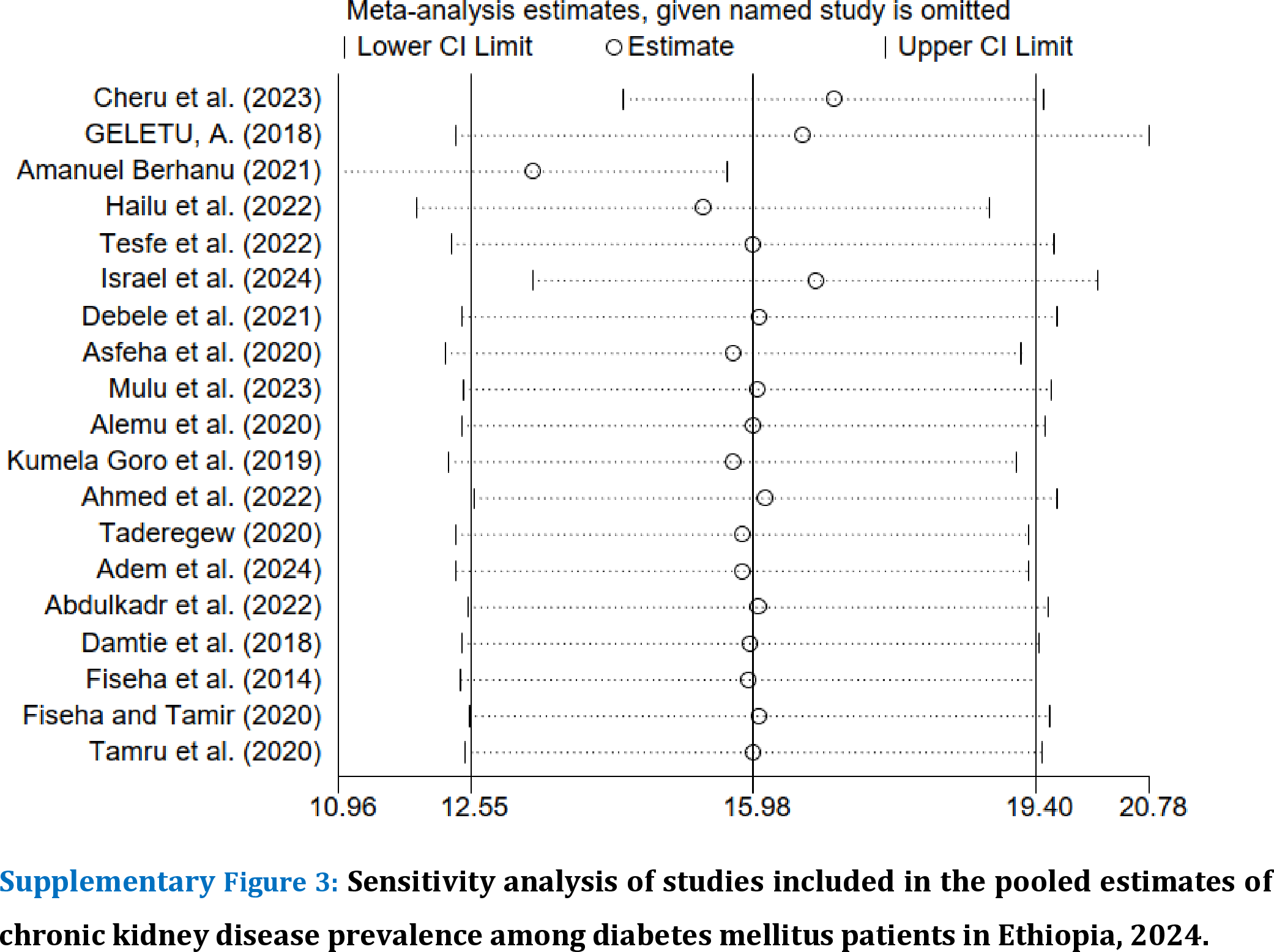
**
